# Obesity: A New Adverse Effect of Antibiotics?

**DOI:** 10.3389/fphar.2018.01408

**Published:** 2018-12-03

**Authors:** Fernando S. Del Fiol, Victor M. Balcão, Silvio Barberato-Fillho, Luciane C. Lopes, Cristiane C. Bergamaschi

**Affiliations:** ^1^Seriema – Evidence Service for Monitoring and Evaluation, University of Sorocaba, Sorocaba, Brazil; ^2^PhageLab – Laboratory of Biofilms and Bacteriophages of UNISO, i(bs)^2^ – Intelligent Biosensing and Biomolecule Stabilization Research Group, University of Sorocaba, Sorocaba, Brazil

**Keywords:** obesity, antibiotics adverse effects, dysbiosis, gut microbiota, antimicrobial

## Abstract

Since the introduction of antibiotics, they have been used freely, with their prescription occurring almost always when they were not necessary. The other major form of contact between humans and antibiotics, now unintentionally, is with the large amount of these drugs in the environment and in our food. The relationship between antibiotic use and the development of obesity has become increasingly evident and apparent in humans, with some authors clearly establishing the relationship between the large-scale use of antibiotics in the past 70 years and the “epidemic” of obesity that has occurred in parallel, almost as an adverse epidemiological effect. In the research effort entertained herein, a correlation between the use and abuse of antibiotics and the onset of obesity was investigated.

## Introduction

The introduction of antibiotics into clinical practice almost 70 years ago was, without a shadow of a doubt, the greatest therapeutic change in medical history, significantly altering mortality and morbidity indicators related to infections. “Magical bullets” have long been considered the Holy Graal for healing infections, but time has shown that we were wrong (Zaffiri et al., 2012).

Since the introduction of antibiotics, they have been used freely, with their prescription occurring almost always when they were not necessary. Recent data from the Centers for Disease Control and Prevention (CDC) shows that, 4 out of 10 children taken to a doctor’s office with a common cold received antibiotics, certainly without necessity (Kronman et al., 2014).

Since their introduction into clinical practice, almost 80 years ago, millions of tons of antibiotics have been produced and used, almost always prophylactically, in cleaning products, cosmetics, etc., The use in veterinary medicine is even more significant, corresponding to 75% of all the antibiotics produced in the American market (Fauci and Marston, 2014). Beyond the exaggerated clinical use in children, we are still exposed to significant amounts of antibiotics in the water we drink (Donoghue, 2003; Yang and Carlson, 2003; Andersson and Hughes, 2014; Venkatesan and Halden, 2014) and even before we are born. A Danish study evaluated about one million of pregnant women and about one-third of them were exposed to one or more systemic treatments with antibiotics during their gestational period (Broe et al., 2014).

Cox and Blaser (2015), in an important study published in 2015, evaluating almost 70% of all pharmaceutical prescriptions made in 2010 in the United States; show that American children, on average up to 2 years of age, received three full medications with antibiotics. Ten full antibiotic medications up to 10 years and around 17 full antibiotic medications until they turned 20 years of age (Cox and Blaser, 2015).

Despite both the abundant literature and information on the inefficacy of antibiotics in fighting viral infections, studies continue to show that the use of antibiotics in combating viral respiratory infections remains high. Ebell and Radke (2015) evaluated ca. 55000 visits of physicians by patients, presumably with ARTI (acute respiratory tract infections). The results showed that 49% of the patients with ARTI received prescriptions containing antibiotics; 8.9% of the patients received within 28 days a second prescription containing one antibiotic, and an astonishing 0.7% of the patients received a prescription with a third antibiotic to treat a viral infection (Ebell and Radke, 2015).

In a study published in Japan, the authors investigated almost 25,000 medical appointments related to upper respiratory tract infections, with no indication of bacterial etiology. The authors reported that antibiotics were prescribed in 60% of the appointments, with third-generation cephalosporins predominating among the antibiotics prescribed for these infections of viral etiology (Higashi and Fukuhara, 2009).

In a study published in 2014, the authors showed that more than half (51%) of the patients with cough/cold diagnoses received antibiotic prescriptions by general practitioners in the United Kingdom in the year 2011. Using the same methodology, the authors of the study demonstrated that in 1999 such percentage was much lower (36%) (Hawker et al., 2014).

Despite all the scientific literature, as well as International associations such as the World Health Organization, being emphatic in the guidelines for decreasing the number of antibiotic prescriptions and, when necessary, prescribing them in a more rational way (Cima, 2014), the numbers and studies continue to show an exaggerated use of these medicines, especially in children.

The other major form of contact we have had with antibiotics, now unintentionally, is with the large amount of these drugs both in the environment and in our food.

It is well known that the use of antibiotics in the production of animal proteins (cattle, pigs, and chickens) has been taking place since the 1940s, dumping thousands of tons of antibiotics into the feed of these animals. In the 1940s, Moore et al. published an article stating that sulfadiazines and aminoglycosides could increase the weight of chickens when incorporated into their diet. These authors concluded that “sulfasuxidine and streptomycin, single or in combination, lead to increased growth responses in chicks receiving our basal diet supplemented with adequate amounts of folic acid” (Moore et al., 1946).

The use of these antibiotics increases the conversion of feed into weight in these animals. The increase in weight of the treated animals may reach from 8 to 15% (Butaye et al., 2003).

For a long time, the increase in both weight and size of animals treated with antibiotics added to their diet was attributed to the decrease in the appearance of subclinical infections, which would favor their growth (Braude et al., 1955; Griffin, 1980; De Wilde, 1984; Elwinger et al., 1998). In fact, what happens is a phenomenon called dysbiosis, characterized by the significant alteration of the intestinal microbiota of these animals, favoring the predominance of microorganisms with greater capacity of metabolization of foods which, as a consequence, will provide a higher amount of calories to the animal, favoring its increase in weight, especially fat (Raoult, 2008a; Thuny et al., 2010; Million et al., 2012). It is important to remember that the classes of antibiotics most used to promote growth in animals are tetracyclines, macrolides, and penicillins, the very same classes used in human beings.

These antibiotics, in small amounts, reach humans either via consumption of the meat of these animals or by the immense amount of such drugs deposited in the soil. The Food and Drug Administration reported that, in 2011, 13500 tons of antibiotics were used for the production of animal proteins through raising cattle, poultry and pigs (Riley et al., 2013). Taking into account that ca. 75% of these antibiotics administered to animals are not absorbed, being eliminated via feces and urine, there are around 10 million kilograms of antibiotics dumped annually into the environment only by the feces and urine of these animals (Chee-Sanford et al., 2009). It is important to remember that antibiotics also reach the environment via spraying of crops (McManus et al., 2002), as well as via hospital wastewaters and overdue medicines that reach our rivers, contaminating the public water supply with small, yet detectable, concentrations of antibiotics (Kulkarni et al., 2017; Szekeres et al., 2017).

At a planetary scale, these numbers are even more worrying. Data from *The State of the World’s Antibiotics 2015* allows to conclude that out of a total of 100000 tons of antibiotics produced worldwide, 65000 tons were used in animal production (Singer et al., 2016).

By making a rough calculation, estimating the world population at 7.2 billion human beings (World Population, 2017) and a worldwide production of antibiotics in the order of 100000 tons (10^11^ g), one obtains as a final result the absurd value of 13.8 g of antibiotic produced per year per inhabitant of our planet.

The exposure to agents that alter our microbiota has increased significantly in the last 80 years, whether as the form of treatments or prophylaxis, or even indirectly via consumption of foodstuff contaminated with these molecules, or even via the immense quantities dumped annually into our environment.

There is no doubt that our microbiota, exposed to 80 years of aggressions through antibiotics, is quite different from the microbiota of our grandparents and our ancestors (Blaser and Falkow, 2009).

Certainly, with a microbiota quite different from that of our ancestors, we have been metabolizing and utilizing our food during the digestive process in a very different way than we did hundreds (or even thousands) of years ago. Are there consequences of this?

## Human Microbiota and Its Role

The human microbiota (called flora, until recently), especially the gut microbiota, has been the subject of hundreds of research studies aiming at shedding light on its role, its composition and the relationships that it establishes with its host (Alexander et al., 2017; Duranti et al., 2017; Koppel and Maini Rekdal, 2017; Mirzaei and Maurice, 2017; Thursby and Juge, 2017).

The intestinal microbiota is composed by ca. 100 trillion cells, including bacteria, fungi, viruses and other eukaryotic species. There are basically five major Phyla: *Firmicutes*, *Bacteroidetes*, *Proteobacteria*, *Verrucomicrobia*, and *Actinobacteria* (Yang and Kweon, 2016). The amount of microorganisms present in the human microbiota exceeds by more than 10 times the number of human cells integrating the human body (Goulet, 2015), and the genes carried by these trillions of microorganisms (called “microbioma”) surpass by more than 100 times the human genome, bringing a symbiotic relationship and of co-existence between the human host and his “guests” (Sanz et al., 2010).

During our intrauterine life, our gastrointestinal tract is totally devoid of microbial life (and thus, practically sterile), although some authors claim that upon swallowing amniotic fluid by the fetus there would be the possibility of forming an intestinal “intrauterine” microbiota (DiGiulio et al., 2008). The microbiota is established by contact. Children born via cesarean birth are, however, deprived of such contact, not contaminating themselves with the mother’s microbiota (Makino et al., 2013).

The human microbiota begins to develop from birth, by vertical transmission, upon contact of the baby with both the vaginal, fecal and cutaneous microbiota, and exposure to breast milk (Reinhardt et al., 2009). It develops with the contact with other family members, also suffering interference from the diet (Backhed et al., 2005), age (Agans et al., 2011), sex(Markle et al., 2013), and geographical location (Grzeskowiak et al., 2012), and suffering also chemical and/or biological interference arising from exposure to antibiotics or probiotics. These interferents alter the microbiota most strongly until late childhood (3 years of age), time at which it stabilizes for the adult life (Vael et al., 2011).

The intestinal microbiota exerts numerous known functions: **(a)** trophic effect upon the intestinal epithelium, favoring the appearance of microvilli, which increases the capacity of nutrient absorption; **(b)** contributes to the homeostasis of the local immune system (Stappenbeck et al., 2002); **(c)** acts on the metabolization of indigestible polysaccharides, favoring the absorption of short chain fatty acids (SCFA) (Turnbaugh et al., 2006); and **(d)** regulates the intestinal transit, affecting the amount of energy absorbed from the diet (Vandeputte et al., 2016). All these functions, especially those related to metabolism and absorption, clearly show the fundamental role that the human microbiota exerts on its host’s weight gain or loss.

Due to having established structure and organic functions, numerous authors already consider the human intestinal microbiota as a new (invisible) organ (Clarke et al., 2014; Pluznick, 2014; Goulet, 2015; O’Callaghan et al., 2016). Toivanen et al. (2001) compared the weight of the human microbiota (1.5 kg) with other organs such as liver (1.5 kg), brain (1.4 kg), lung (0.84 kg), and kidney (0.27 kg), thus shedding light upon the importance that this new (although invisible) organ represents to the human organism.

## Human Microbiota and Metabolism

One of the major (and perhaps the most important) functions played by the intestinal microbiota is the metabolism of nutrients. In order to exert their function of withdrawing energy from the foodstuff consumed by their hosts, the microbiota produces enzymes such as glycoside hydrosilases and polysacharide lyases with the role of withdrawing energy from inaccessible foods (Villanueva-Millan et al., 2015).

The role of the microbiota in the uptake of energy from food, by transforming it into absorbable fat, has been elucidated in a more convincing manner by studies using germ-free animals. Regular animals, with normal microbiota, subjected to the same diet as their “germ-free” counterparts, accumulated more fat when compared to each other, contributing to the accumulation of fat in the former animals. The authors concluded their study by stating that “*Our findings suggest that the gut microbiota is an important environmental factor that affects energy harvest from the diet and energy storage in the host*” (Backhed et al., 2004).

Indigestible oligosaccharides, such as oligofructose (inulin), are converted by the gut microbiota into monosaccharides which are then converted to SCFA (acetate, propionate, and butyrate), being then easily absorbed (Villanueva-Millan et al., 2015). The acetate and propionate formed are used by the liver for lipogenesis and gluconeogenesis. Butyrate has its role as energy source for colonic epithelial cells (Janssen and Kersten, 2015). The conversion of polysaccharides by microorganisms from the gut microbiota into SCFAs has been implicated as the factor responsible for the increased uptake of fat and concomitant obesity in human hosts (Greenblum et al., 2012; Woting and Blaut, 2016).

For many years obesity has been treated as a pandemic, with constant increases in the prevalence among adolescent and children, being considered a real threat to the human condition.(Katz, 2016) Worldwide, there are more than 2 billion individuals considered to be overweight or obese. The prevalence rate for these conditions increased 27.5% for adults and 47.1% for children between 1980 and 2013 (Ng et al., 2014).

The condition of obesity originates in multifactorial situations, including genetic (van der Klaauw and Farooqi, 2015), physiological (leptin modulation) (Bajzer and Seeley, 2006), heredity (Farooqi and O’Rahilly, 2006), socio-cultural behaviors among populations; such as geography, food preferences, physical activity, gender, age, etc (Williams et al., 2015).

Over the last few years, and because of all the personal and public health disorders caused by obesity, numerous studies have been published showing the relevant role of the gut microbiota in the development of obesity (Reinhardt et al., 2009; Zhang et al., 2009; Scarpellini et al., 2010; Boroni Moreira et al., 2012; Bruce-Keller et al., 2015; Cox and Blaser, 2015; Duranti et al., 2017; Isolauri, 2017; Raoult, 2017).

Some studies relate the change in the proportion of the phyla Firmicutes:Bacteroidetes as responsible for the change of weight gain/loss. Such studies indicate that the increase of the phylo Firmicutes in relation to the Bacteroidetes would be responsible for the increase in the absorption of calories from food, supplying larger amounts of fat to the host with concomitant increase in both its weight and fat mass (Ley et al., 2005; Compare et al., 2016; Koliada et al., 2017; Kvit and Kharchenko, 2017; Riva et al., 2017; Shang et al., 2017).

In this way, any disturbance in the microbiota (dysbiosis) may alter the Firmicutes:Bacteroidetes ratio, leading to a higher amount of Firmicutes and concomitant higher absorption of fats, increasing both the weight and the amount of fat of the host. These microbiota changes (dysbiosis) are caused by treatments with antibiotics, especially when administered before the first 3 years of life, even in small amounts and during short periods of time (Riley et al., 2013; Lu and Ni, 2015; Duranti et al., 2017; Isolauri, 2017).

## Antibiotics and Obesity

Results from controlled epidemiological studies carried out in the last 6 years (see Table 1) involving 368360 children (sum of all studies) have always associated early-life (or even intrauterine) exposure to antibiotics with weight gain or BMI increase. It is important to emphasize that such association relates not the use of the antibiotic directly with obesity, but rather the antibiotic being the cause of the alteration of the microbiota (dysbiosis) and concomitant increase in the energy intake from the diet and consequent obesity.

**Table 1:** Epidemiological studies in children in the last 6 years, showing the relationship between the use of antibiotics and obesity.

Title of the study	Year	Subjects of the study	Final result(s) gathered	Reference
Childhood overweight after establishment of the gut microbiota: the role of delivery mode, pre-pregnancy weight and early administration of antibiotics.	2011	28354 mother-child	Antibiotics in infancy influences the risk of overweight in later childhood	Ajslev et al., 2011
Infant antibiotic exposures and early-life body mass.	2013	11532 children	Exposure to antibiotics during the first 6 months of life was associated with increases in body mass.	Trasande et al., 2013
Antibiotic treatment during infancy and increased body mass index in boys: an international cross-sectional study.	2014	74946 children	Exposure to antibiotics during the first 12 months of life is associated with a small increase in BMI in boys aged 5–8 years	Murphy et al., 2014
Infant antibiotic exposure and the development of childhood overweight and central adiposity	2014	1047 children	Antibiotic use in the first year of life was associated with overweight	Azad et al., 2014
Association of antibiotics in infancy with early childhood obesity.	2014	64580 children	Repeated exposure to broad-spectrum antibiotics was associated with early childhood obesity	Bailey et al., 2014
Prenatal exposure to antibiotics, cesarean section and risk of childhood obesity.	2015	436 mother-child dyads	Exposure to antibiotics in the second or third trimester of pregnancy were associated with higher risk of childhood obesity.	Mueller et al., 2015
Prenatal exposure to systemic antibacterials and overweight and obesity in Danish schoolchildren: a prevalence study.	2015	9886 children	Prenatal exposure to systemic antibacterials was associated with an increased risk of overweight and obesity at school age	Mor et al., 2015
Antibiotic exposure in infancy and risk of being overweight in the first 24 months of life.	2015	6114 boys and 5948 girls	Antibiotic exposure before 6 months was associated with increased body mass	Saari et al., 2015
Early Life Antibiotic Exposure and Weight Development in Children.	2016	979 children	Repeated exposure to antibiotics early in life, especially β-lactam agents, is associated with increased weight and height.	Mbakwa et al., 2016
Antibiotic Use and Childhood Body Mass Index Trajectory.	2016	142824 children	Body Mass Index increase	Schwartz et al., 2016
Administration of Antibiotics to Children Before Age 2 Years Increases Risk for Childhood Obesity.	2016	21714 children	Administration of 3 or more courses of antibiotics before age of 2 years was associated with an increased risk of early childhood obesity	Scott et al., 2016


Studies in germ-free animals that received a microbiota transplant and did not receive any antibiotics whatsoever, developed obesity without contact with antibiotics, thus proving that gut microbiota plays a fundamental role in the accumulation of fat. The antibiotic serves only to alter it (Backhed et al., 2004; Turnbaugh et al., 2006; Goodman et al., 2011; Kimura et al., 2013; Cox et al., 2014; Gerard, 2016). Studies carried out using regular animals (non-germ-free) that received antibiotics point to the same direction, increasing the weight of the animals due to changes in their gut microbiota (Cho et al., 2012; de Sa Del Fiol et al., 2014; Khan et al., 2016; Marciano et al., 2017).

The relationship between the use of antibiotics and the development of obesity has become increasingly more evident and apparent in human beings. Some authors have clearly established the relationship between the use of antibiotics in large-scale in the past 70 years and the “epidemic” of obesity that has occurred in parallel, almost as an adverse epidemiological effect (Ternak, 2005; Raoult, 2008b; Riley et al., 2013).

Data gathered from the CDC on Antibiotic Prescriptions Dispensed in United States. Community Pharmacies per 1000 inhabitants in 2014 show, on average, 835 antibiotic prescriptions dispensed per 1000 inhabitants (CDC, 2014). Such data, separated for the 50 individual American states, was compared with the obesity indicators of those same states (data also provided by the CDC) (CDC, 2015). The data on antibiotic prescriptions (n/1000 people) and indication of obesity (%) are displayed and compared in Table 2.

**Table 2:** Percentage of obesity and antibiotic prescription rate in all American States. Data from 2014, sourced from the Centers for Disease Control and Prevention (CDC).

			Antibiotic prescription/	Antibiotic
American state	Obesity (%)	Obesity rank	1000 inhabitants	prescription rank
Louisiana	36.2	1	1177	4
Alabama	35.6	2	1124	7
Mississippi	35.6	3	1222	3
West Virginia	35.6	4	1285	1
Kentucky	34.6	5	1262	2
Arkansas	34.5	6	1155	6
Kansas	34.2	7	992	10
Oklahoma	33.9	8	966	11
Tennessee	33.8	9	1162	5
Missouri	32.4	10	936	15
Texas	32.4	11	899	21
Iowa	32.1	12	1001	9
South Carolina	31.7	13	927	16
Nebraska	31.4	14	1045	8
Indiana	31.3	15	951	13
Michigan	31.2	16	925	17
North Dakota	31	17	853	26
Illinois	30.8	18	853	25
Georgia	30.7	19	841	28
Wisconsin	30.7	20	745	34
South Dakota	30.4	21	901	19
North Carolina	30.1	22	861	24
Oregon	30.1	23	570	49
Maine	30	24	720	37
Pennsylvania	30	25	886	23
Alaska	29.8	26	502	50
Ohio	29.8	27	965	12
Delaware	29.7	28	938	14
Virginia	29.2	29	799	29
Wyoming	29	30	778	32
Maryland	28.9	31	796	30
New Mexico	28.8	32	714	38
Idaho	28.6	33	693	42
Arizona	28.4	34	740	35
Florida	26.8	35	729	36
Nevada	26.7	36	709	40
Washington	26.4	37	600	47
New Hampshire	26.3	38	702	41
Minnesota	26.1	39	710	39
Rhode Island	26	40	893	22
New Jersey	25.6	41	903	18
Connecticut	25.3	42	847	27
Vermont	25.1	43	639	45
New York	25	44	900	20
Utah	24.5	45	783	31
Massachusetts	24.3	46	769	33
California	24.2	47	570	48
Montana	23.6	48	648	43
Hawaii	22.7	49	644	44
Colorado	20.2	50	625	46


**FIGURE 1:**
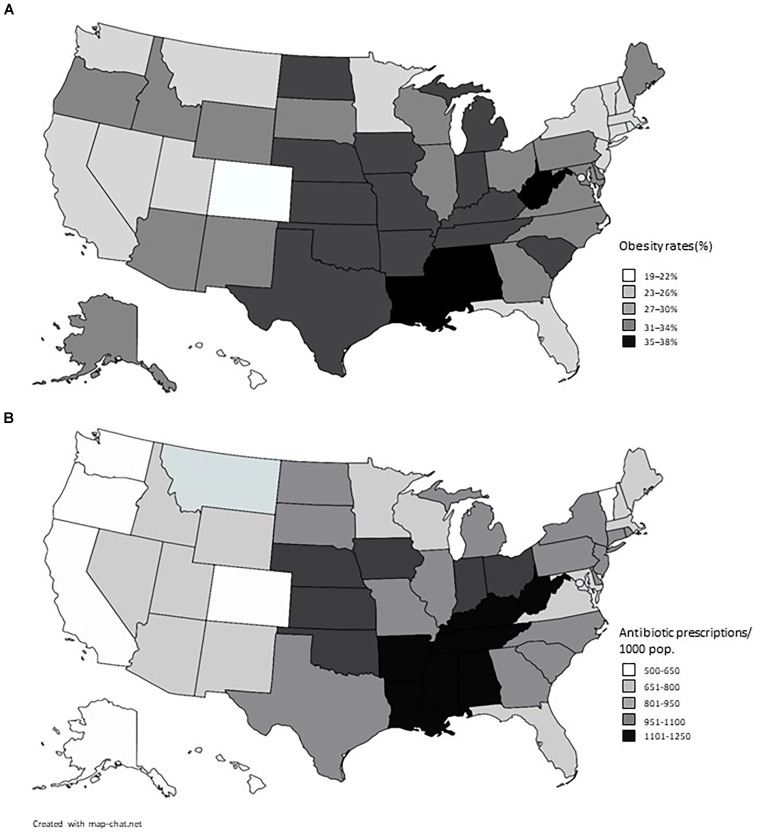
American maps showing rates of obesity **(A)** and prescription of antibiotics **(B)** in the American States. Data from 2014, sourced from the Centers for Disease Control and Prevention (CDC).

The results displayed in Table 2 exhibit a great similarity between the use of antibiotics in the United States and the indicators of obesity. When Pearson’s correlation coefficient is applied, a result of 0.76 is obtained which is considered a positive and strong correlation, meaning that the higher the use of antibiotics the higher the rate of obesity at that place (Mukaka, 2012). The indexes of obesity (CDC, 2015) and of use of antibiotics (CDC, 2014) are plotted in Figure 1.

**FIGURE 2:**
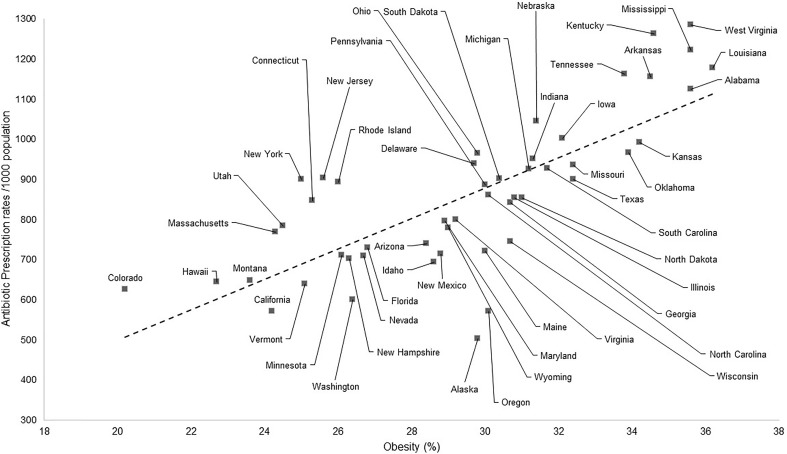
Correlation between the antibiotic prescription rates (per 1000 inhabitants) and indicators of obesity (%). Data from 2014, sourced from the CDC.

In the same way, however, by illustrating the data in a graphical fashion one can perceive much similarity between the data on obesity and the use of antibiotics, shown on the maps of the United States (see Figure 2).

## Conclusion

The answer to the title of this short review paper seems to be YES. The scientific community has reached a consensus on the relationship between antibiotics and obesity, showing beyond any doubt that the use of antibiotics causes dysbiosis to varying degrees, especially in children (with less than 3 years of age), which can trigger an increased energy intake from the diet with concomitant increase of weight.

There is no doubt whatsoever that antibiotics have been, and still are, drugs with vital importance in the fight against bacterial infections. However, the need for prudent and rational use of antibiotics is more and more clear so as to avoid both collective health problems (increase in bacterial resistance to these antimicrobial drugs) and individual health problems (increase in weight).

The alternative for replacing the microbiota with probiotics seems to be an important strategy for the safe use of antibiotics to fight bacterial infections in children, without the associated risk of dysbiosis and concomitant increase in body weight.

It is the vital role of the health-care professionals to orient both themselves and their patients toward the correct and prudent use of antibiotics, and to wait until further scientific studies enlighten even more the relationship between antibiotics, dysbiosis, and obesity.

## Author Contributions

FDF, VB, SB-F, LCL, and CB designed the study, involved in the selection and reading of the review articles, prepared the manuscript, and discussed the results and conclusion.

## Conflict of Interest Statement

The authors declare that the research was conducted in the absence of any commercial or financial relationships that could be construed as a potential conflict of interest.
